# The development and characterization of a stable Coxsackievirus A16 infectious clone with Nanoluc reporter gene

**DOI:** 10.3389/fmicb.2022.1101850

**Published:** 2023-01-10

**Authors:** Rui Yu, Min Wang, Lizhen Liu, Jingjing Yan, Jun Fan, Xiaohong Li, Miaomiao Kang, Jianqing Xu, Xiaoyan Zhang, Shuye Zhang

**Affiliations:** ^1^Shanghai Public Health Clinical Center and Institutes of Biomedical Sciences, Fudan University, Shanghai, China; ^2^Clinical Center for Biotherapy, Zhongshan Hospital, Fudan University, Shanghai, China

**Keywords:** Coxsackievirus A16, infectious clone, Nanoluc, high-throughput screening, neutralizing antibody

## Abstract

Coxsackievirus A16 (CA16) belongs to the *Human Enterovirus A species*, which is a common pathogen causing hand, foot, and mouth disease in children. Currently, specific vaccines and drugs against CA16 are unavailable, and there is an unmet need to further understand the virus and invent effective treatment. Constructing a CA16 infectious clone with a reporter gene will greatly facilitate its virological studies. Here, we first reported the construction of a CA16 infectious clone (rCA16) whose progeny is highly replicative and virulent in suckling mice. On the basis of rCA16, we further inserted a NanoLuc (Nluc) reporter gene and made the rCA16-Nluc clone. We found that the Nluc gene in rCA16-Nluc is stable during continuous growing in Vero cells and thus allowed detection of a steady luciferase signal in rCA16-Nluc-infected Vero cells over 10 passages. Its application in antivirals characterization and high-throughput screening is exemplified by measuring IC_50_, CC_50_, and selection index of guanidine hydrochloride, ribavirin, chloroquine, and ammonium chloride against CA16. Finally, we showed that rCA16-Nluc based assay greatly simplified the CA16 neutralizing antibody tests. Thus, these two CA16 infectious clones will be robust tools for future enterovirus studies and antivirals development.

## Introduction

Coxsackievirus A16 (CA16), is a member of species *Enterovirus A*, genus *Enterovirus*, family *Picornaviridae* (Baggen et al., 2018). As a single-stranded, positive-strand RNA virus, CA16 is ~7.4 kb in length and contains a 5′UTR, an open reading frame (ORF), a 3′UTR and a polyadenosine (polyA) tail (Liu et al., 2011). Its ORF encodes one polyprotein and is cleaved into VP4, VP3, VP2, and VP1 (viral structural proteins) and 2A, 2B, 2C, 3A, 3B, 3C, and 3D (viral non-structural proteins; Chua et al., 2022).

Hand, foot, and mouth disease (HFMD) mainly infect infants and young children. Generally, HFMD is mild and self-limited. However, severe diseases may include paralysis, myocarditis, and even death (Esposito and Principi, 2018). CA16 is one of the most prevalent HFMD pathogens in the Asia-Pacific region, besides EV71 (Liu W. et al., 2014; Liu et al., 2015). At present, only the inactivated virus vaccine against EV71 is commercially available (Shimizu, 2021), and there are no vaccines or drugs against other enteroviruses, including CA16 (Chen et al., 2014; Mao et al., 2014). Therefore, there is an unmet need to further understand different enteroviruses and invent effective treatments.

A reporter virus is valuable for virological studies and antivirals development (Cruz-Cardenas et al., 2022). Although a CA16 infectious clone with a eGFP reporter has been reported (Deng et al., 2015), the eGFP in enterovirus is usually unstable and may quickly disappear upon a few passages. In addition, the quantitative analysis of the eGFP reporter is cumbersome and not high-throughput. In contrast, the luciferase reporter is more suitable for rapid and high-throughput detection. An EV71 reporter virus carrying Gaussia luciferase (Gluc) was previously reported, but the short half-life of Gaussia luciferase limits its application in high-throughput screening (Xu et al., 2015). To overcome this, EV71 with a novel luciferase Nanoluc (Nluc) reporter was recently developed (Yang et al., 2021). Nluc has the advantages of smaller size and higher luminescence than Gluc, and is more suitable for high-throughput screening.

Here, we successfully constructed two infectious clones of a clinical CA16 isolate (Genebank: OP293089) and characterized the rCA16 and rCA16-Nluc virus, respectively. Our data indicated that both recombinant viruses showed comparable growth characteristics as the parental CA16 (pCV16) virus *in vitro*. Intraperitoneal inoculation of both pCA16 and rCA16 in one-week-old lactating mice led to severe multiple-organ/tissue damage and was 100% lethal within 6 days, indicating high pathogenicity of this CA16 strain. We also found that the Nluc gene in rCA16-Nluc is genetically stable over ten passages in cultured cells without losing the reporter gene. Moreover, by measuring fluorescence intensity, we have shown that the application of rCA16-Nluc has simplified testing for antivirals and neutralizing antibodies, and is fully compatible with high-throughput screening platforms, suggesting a promising application potential.

## Materials and methods

### Cells and virus

African green monkey kidney (Vero) cells used in this study were maintained in Dulbecco’s modified Eagle’s medium (DMEM) supplemented with 10% FBS, 100 U/mL of penicillin, and 100 μg/mL of streptomycin at 37°C in presence of 5% CO_2_. The CA16 strain OP293089 was isolated in the Biosafety Laboratory of Shanghai Public Health Clinical Center.

### Construction of plasmids and validation sequencing

The CA16 viral RNA was extracted with QIAamp Viral RNA Mini Kit (Qiagen, Germany) and reversed transcribed with oligo dT and HiScript II 1st Strand cDNA Synthesis Kit (Vazyme, China). Using the viral cDNA as the template, three viral fragments: P1, P2, and P3 were amplified. Then, the P1 fragment was cloned in the pcDNA vector (pcDNA-P1), and P2 and P3 fragments were cloned in the pUC57 vector (pUC57-P2+P3). Next, P1 and P2+P3 two fragments were ligated with the pSVA vector to form the infectious DNA clone of CA16 (rCA16). To get the CA16 infectious clone with a Nluc reporter gene (rCA16-Nluc), the Nluc sequence was inserted between 5′UTR and VP4. The genetic stability of Nluc gene was examined by PCR amplification and sanger sequencing, the PCR parameters and primers for Nluc PCR/sequencing were described in the Supplementary Tables 1, 2. The detailed process of the clone construction and the primers used were described in the Supplementary File and Supplementary Table 3.

### *In vitro* transcription and transfection

To generate viral mRNA, the vectors of rCA16 and rCA16-Nluc infectious clone was first linearized by Sal I restrictive enzyme (NEB, The United States), then purified as the template for *in vitro* transcription using T7 Transcription Kit (Novoprotein, China) according to the manufacturer’s instruction. Next, the purified viral mRNA was transfected to Vero cells by Lipo3000 (Thermo Fisher Scientific, The United States) and incubated for 6 h at 37°C before the medium was changed to fresh complete medium.

### Western blot

Cells for WB detection were harvested and lysed in 1 × SDS loading buffer (NCM Biotech, China) and heated for 10 min at 100°C. Next, cell lysates were loaded and separated by 10% SDS-PAGE gel and transferred to PVDF membrane. Then, the PVDF membrane was blocked with 5% milk, and stained with the viral 2C monoclonal antibody (Youke, China; Liu et al., 2021) and then HRP-Mouse secondary antibody (Yeason, China) to probe viral protein.

### Immunofluorescence assay

Vero cells were seeded on coverslips overnight and infected with the virus at MOI = 1, for another 8 h. Then, the infected cells were washed with cold PBS twice and fixed with 4% paraformaldehyde (Sigma, St Louis, United States) for 15 min. After permeabilization with 0.05% Triton X-100 in 2% FBS, the cells were stained with the 2C or 3D antibody for 1 h. Next, cells were washed with PBS twice and incubated with secondary antibodies AlexoFluo594 for 30 min, followed by washing three times. Finally, cells were stained with DAPI and analyzed using EVOS^®^ FL Color Imaging Systems (Life technology, The United States).

### Plaque assay

The virus was 10-fold serially diluted and added to Vero cells in a 12-wells plate, incubated for 2 h at 37°C, then washed with PBS twice, and loaded with 2 × DMEM (4% FBS) and an equal volume of Avicel (IMCD, China). Afterwards, the 12-wells plate was incubated at 37°C for another 3 days. Then, the plate was fixed with 4% paraformaldehyde for 2 h and stained with 1% crystal violet for another 2 h.

### Antiviral assay

For testing virus replication inhibitors: 2.5 × 10^4^ Vero cells were seeded in 96-well plates and incubated overnight, before infection with rCA16-Nluc virus at MOI = 0.1. Two hours post-infection, culture medium was replaced with fresh DMEM medium containing different concentrations of GuHCl or ribavirin for 24 h. Next, luciferase activity was measured according to the manufacturer’s instructions.

For testing virus entry inhibitors: 2.5 × 10^4^ Vero cells were seeded in 96-well plates and incubated overnight, then the culure medium were replaced with DMEM medium containing different concentrations of chloroquine or NH_4_Cl for 2 h at 37°C. Afterwards, the cells were infected with rCA16-Nluc virus at MOI = 0.1 for 2 h; then the culture medium was replaced with fresh maintaining DMEM medium for 24 h before measuring the luciferase activity.

### Cell viability analysis

After being treated with various concentrations of chemical inhibitors, cell viability was analyzed by Cell Counting Kit-8 (CCK-8, Dojindo Laboratories, Japan). Specifically, 3 * 10^4^ Vero cells were seeded in 96-well plates and cultured overnight. Then various concentrations of GuHCl, ribavirin, NH_4_Cl, and chloroquine in 2%FBS DMEM were added as triplicates per condition. The next day, the CCK-8 solution was added to each well and incubated at 37°C for 1 h, after which the absorbance at 460 nm was measured (Cytation5, Biotek, The United States).

### Animal assay

Twenty-five 7-day-old C57/B6 suckling mice were randomly divided into five groups, including two pCA16 infection groups (*n* = 5), two rCA16 infection groups (*n* = 5), and one PBS control group (*n* = 5). Mice of the infection group were inoculated with 10^5^ TCID_50_ pCA16 and rCA16 viruses *via* intraperitoneal routes, while the control mice were given the same volume of PBS. The body weight of mice was followed each day post-infection. For histology and viral characterization, one group of control, pCA16 and rCA16 mice were sacrificed at 4 days after infection. The brain, thigh muscle, lung, and heart tissues were harvested for viral RNA measurement by q-PCR or Hematoxylin and Eosin (HE) staining for tissue damage evaluation.

### Luciferase based viral neutralization assay

For generation of neutralizing antibodies, five 6–8 weeks old C57/B6 mice were challenged (i.p) with 10^5^ TCID_50_ CA16 viruses twice at a 2-week interval, and the control mice were given the same volume of PBS. Serum samples were collected through the ophthalmic vein in the second and fourth weeks after the primary immunization.

To inactivate complement, the collected sera were heated at 56°C for 30 min, and then were 2-fold serially diluted (1:50–1:25,600) in DMEM and incubated with an equal volume of 10^3^ TCID_50_ rCA16-Nluc at 37°C for 2 h. Uninfected cells as a negative control, and sera from the PBS group as the positive control. The virus-sera mixtures were added to the 96-wells plate where 2.5 × 10^4^ Vero cells/well were seeded overnight, for a 24 h-incubation at 37°C. Afterwards, the luciferase activities were measured. The nAb titers are derived from the highest dilution index to achieve a 50% reduction of the relative luciferase activity. In addition, all the immunized sera were also confirmed by the CPE-reduction observation.

### Statistical analysis

All the viral RNA copies, luciferase assay data were obtained from at least three repeated experiments. The data were analyzed using Prism 7.0 software (GraphPad, United States) and are presented as the means ± SEM. Statistical significance between the two groups was determined by unpaired two-tailed Student’s *t*-test. Differences were considered to be not significant (ns), *p* > 0.05; *, *p* < 0.05; **, *p* < 0.01; ***, *p* < 0.001; ****, *p* < 0.0001.

## Results

### Construction of an infectious clone of Coxsackievirus A16

**Figure 1:**
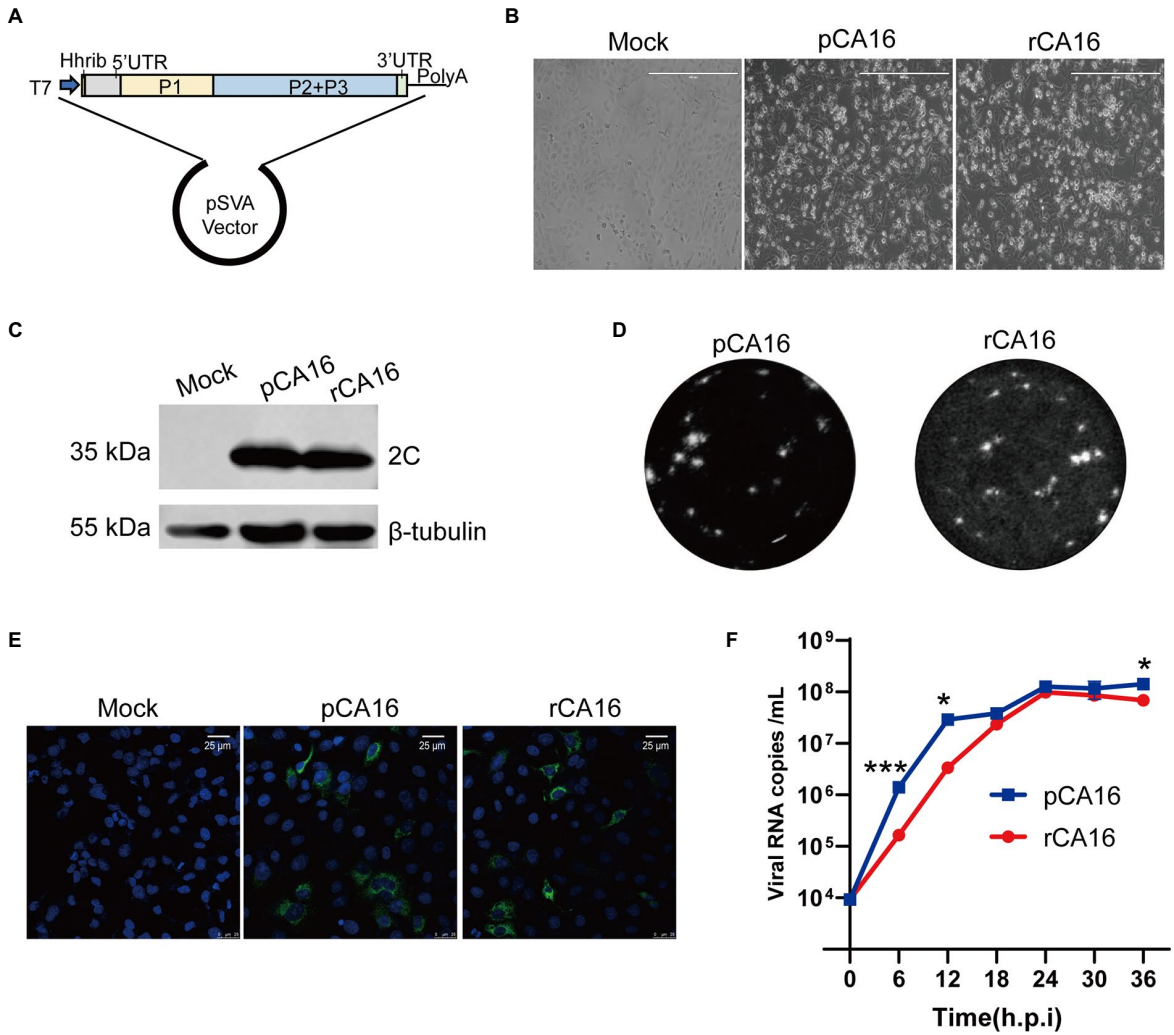
Construction and characterization of the rCA16 infectious clone. **(A)** The complete CA16 cDNA clone was inserted into the pSVA backbone, with a T7 promoter and a hammerhead ribozyme in the front. **(B)** Vero cells were infected with parental CA16 (pCA16) and rCA16 (MOI = 1) and the cytopathic effect (CPE) was examined 24 h after infection. **(C)** Twenty-four hours after the viral infection, pCA16 and rCA16 infected Vero cells were collected for the immunoblot of the viral 2C protein. **(D)** Morphology of pCA16 and rCA16 viral plaque in Vero cells. **(E)** Vero cells were infected with pCA16 and rCA16 (MOI = 1) for 6 h, then the cells were fixed and stained with an anti-3D primary antibody, followed by a staning of Alexa Flour-488 conjugated secondary antibody. DAPI was used for nuclei visualization (Green, 2C; Blue, nuclei). **(F)** The one-step growth curve of pCA16 and rCA16. Viruses infected Vero cells (MOI = 0.1) were harvested at 6, 12, 18, 24, 30 and 36 h after infection for viral RNA quantitation, which peaked at 24 h after infection. Statistical differences were determined using t-test (*P < 0.05; **P < 0.01; ***P < 0.001; ****P < 0.0001).

The CA16 strain OP293089, belonging to the B1b subtype (Supplementary Figure 1) was isolated by the Biosafety Laboratory of Shanghai Public Health Clinical Center. To construct the CA16 infectious clone, the viral RNA was extracted, reverse transcribed to single-strand cDNA, and cloned into pSVA vector which contains a T7 promotor as shown in Figure 1A. We generated viral mRNA from the infectious clone by *in vitro* transcription, and found that the recombinant CA16 viruses (rCA16) could effectively infect Vero cells and cause significant cytopathogenic effect (CPE) at 24 h post-infection (Figure 1B). Western blot using the antibody against viral 2C protein showed the presence of a 35 kDa band, indicating the viral 2C protein was produced (Figure 1C). Besides, an immunofluorescence assay was used to detect the expression of viral 3D protein, showing specifically positive signals in cells infected by rCA16 and pCA16 (Figure 1E). Shown in Figure 1D, rCA16 produced plaques similar to pCA16 in both morphology and numbers. To further characterize the rescued rCA16 viruses, we compared the growth characteristic of rCA16 and pCA16 in Vero cells, both viruses proliferated rapidly after infecting the cells and reaching the plateau level at 24 h post-infection (Figure 1F). Together, these data suggest that we have successfully rescued a rCA16 clone which showed comparable virological characteristics as its parental virus.

### Pathogenicity of the rCA16 virus in lactating mice

**Figure 2:**
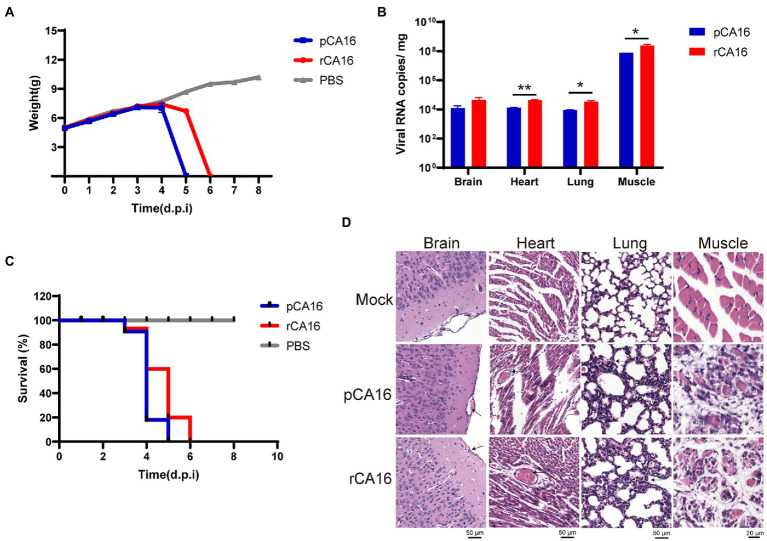
Virulence of the pCA16 and the rescued rCA16. One-week-old lactating mice were challenged intraperitoneally (i.p.) with 10^5^ TCID_50_ of pCA16 and rCA16. Mice in the control group were given an equal volume of PBS. **(A)** Changes in body weight in different groups. **(B)** The survival curve of the infected mice. **(C)** Viral RNA loads in the brain, heart, lungs and muscles 4 days after infection. **(D)** The pCA16 and rCA16 infection caused leptomeningeal hemorrhage, thickened alveolar walls, and ruptured thigh and myocardial muscles. Statistical differences were determined using t-test (*P < 0.05; **P < 0.01; ***P < 0.001; ****P < 0.0001).

Enteroviruses usually infect young children. To examine the virulence and pathogenicity of rCA16, we challenged the one-week-old C57/B6 neonatal mice with 10^5^ TCID_50_ rCA16 and pCA16, respectively. As shown in Figure 2A, the body weight of mice in the PBS group increased gradually, whereas those in rCA16 and pCA16 groups lost weight from 3 to 4 days after infection and also developed paralysis of the hind limb (Supplementary Figure 2). Severe pathogenicity is indicated by the survival curve, mice infected with rCA16 and pCA16 started dying on day 3 and reached 100% mortality after 5–6 days (Figure 2B). We analyzed the viral loads in various tissues and found that the muscle had the highest viral load, up to 10^8^ viral copies/mg, followed by the brain, heart, and lung tissues (Figure 2C), indication of highly productive viral replication in the infected mice. Severe tissue damages were shown by HE staining (Figure 2D). Infection with rCA16 and pCA16 caused hemorrhagic inflammation of the brain and heart, significant thickening of the alveolar wall, and obvious fragmentation of muscle fibers.

Together, our data showed that this CA16 strain and its recombinant virus are highly pathogenic in one-week-old mice, which provides a convenient pathogenic murine model for enterovirus infection.

### Construction of an infectious clone of Nluc-expressing Coxsackievirus A16

**Figure 3:**
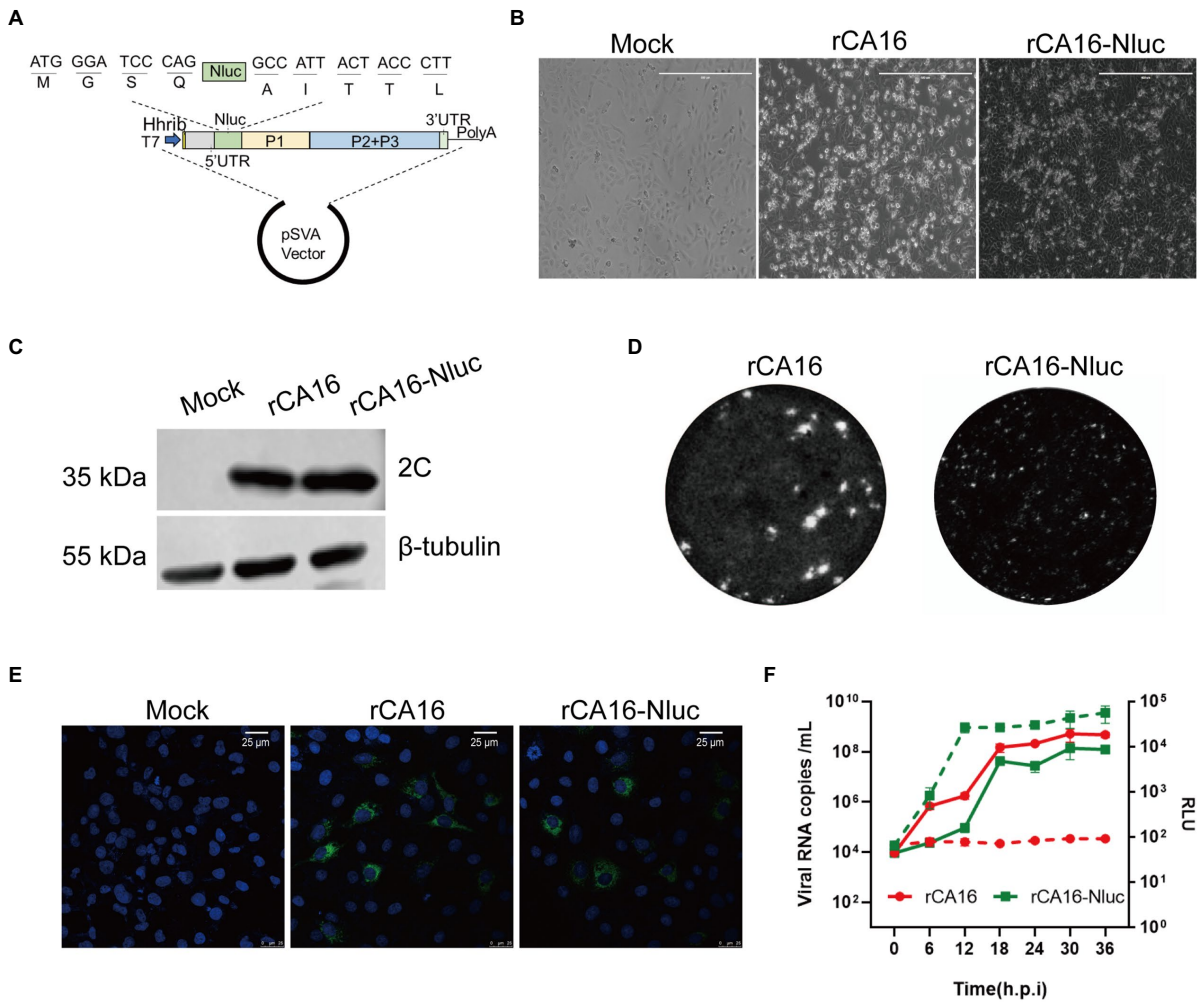
Construction and characterization of the rCA16-Nluc infectious clone. **(A)** The Nluc gene and the 2A proteinase cleavage site (AITTL) were inserted in the rCA16 infectious clone. **(B)** Vero cells infected with rCA16 and rCA16-Nluc produced the cytopathic effect (CPE) 24 h after infection. **(C)** The viral 2C protein and β-tubulin were immunoblotted from rCA16 and rCA16-Nluc infected Vero cells. **(D)** Morphology of rCA16 and rCA16-Nluc viral plaque in Vero cells. **(E)** Vero cells were infected with rCA16 and rCA16-Nluc (MOI = 1) for 6 h, then the cells were fixed and stained with an anti-3D primary antibody, followed by a staning of Alexa Flour-488 conjugated secondary antibody. DAPI was used for nuclei visualization (Green, 2C; Blue, nuclei). **(F)** The one-step growth curve and relative luminescence intensity (RLU) of rCA16 and rCA16-Nluc. Viruses infected Vero cells (MOI = 0.1) were harvested at 6, 12, 18, 24, 30, and 36 h after infection for viral RNA and RLU measurement (The solid line, growth of viral RNA; The dotted line, growth of RLU).

Reporter viruses are useful tools to study viral pathogenesis and screen antiviral drugs. We next constructed a CA16 infectious cDNA clone with a Nanoluc luciferase reporter gene (Nluc). Nluc was inserted following 5′UTR and fused with the viral 2A cleavage site (AITTLG) before the VP4 protein (Figure 3A). Then the Nluc-expressing infectious clone was *in vitro* transcripted into viral mRNA to produce the recombinant CA16 reporter viruses (rCA16-Nluc) by transfection in Vero cells. Vero cells infected with rCA16 and rCA16-Nluc showed significant CPE 24 h after infection (Figure 3B), although the plaques of Nluc-virus are indeed smaller than the rCV16 (Figure 3D), in line with other earlier reports on engineered enteroviruses. Using immunoblotting and immunofluorescence, we detected the expression of viral 2C and 3D proteins at 24 h post-infection in Vero cells (Figures 3C,E). Furthermore, rCA16-Nluc viruses showed similar growth patterns of viral RNAs with rCA16 (Figure 3F). More importantly, besides the viral RNA copies, we also found that luciferase activity increased exponentially following the early stage of infection and reached plateau levels from 12 h post rCA16-Nluc infection, showing a similarly growing kinetics with viral RNAs, whereas rCV16 infection produced only background luciferase signals (Figure 3F). Finally, we also demonstrated that the one-week-old mouse infected with rCA16-Nluc can be monitored through *in vivo* bioluminescence imaging (Supplementary Figure 3). Together, our data indicate that the insertion of Nluc reporter gene only slightly affects the fitness of rCA16, and that the luciferase activity from rCA16-Nluc closely correlates with its viral replication activities.

### Validation of the stability of rCA16-Nluc

**Figure 4:**
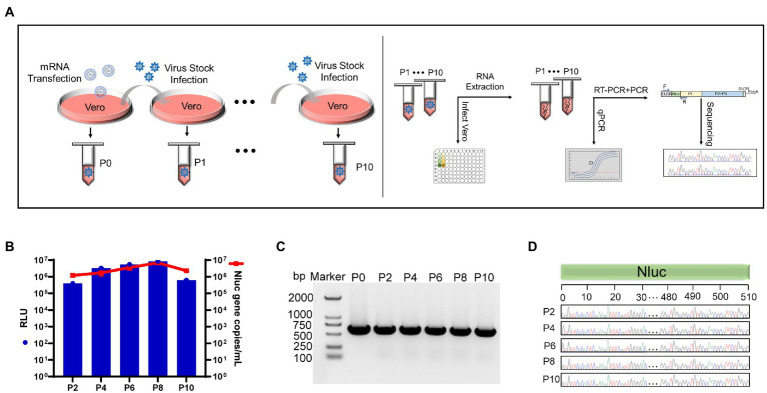
The Nluc insertion is stable in the rCA16-Nluc virus. **(A)** Illustrates the rCA16-Nluc stability test procedure. The left panel shows the series collection of rCA16-Nluc over 10 passages (from P0 to P10) in the Vero cells. The right panel shows the test for luciferase activity and Nluc gene in each viral passage. **(B)** Vero cells were infected by series passages (as indicated) of rCA16-Nluc. Luminescence (RLU value, blue bars) and Nluc gene copies (red line and square) from infected cells were measured. **(C)** The Nluc gene was amplificated by RT-PCR using total RNAs from rCA16-Nluc infected cells as the template, and analyzed by agarose gel. **(D)** Shows the Sanger sequencing of the Nluc gene from different passages of rCA16-Nluc. For better visualization, only nucleotides 0–30 and 480–510 of the Nluc gene are displayed.

The genomic stability of genetically engineered enteroviruses is challenging and unpredictable. To ensure the stability of rCA16-Nluc, the rescued virus was continuingly passaged and monitored in Vero cell cultures, as indicated in Figure 4A. We measured the luciferase activity and quantified Nluc gene copies from passages of P2, P4, P6, P8, and P10 (Figure 4B). Indeed, we found that the gene and activity of the Nluc reporter were maintained at constant levels throughout these extended cultures. We then analyzed the size of the Nluc gene inserts through PCR. As indicated in Figure 4C, the amplified products from P0 to P10 showed the same expected size, indicating no deletion of Nluc gene during the continuous passages. Furthermore, Sanger sequencing of the PCR products verified that the sequence of Nluc gene from P0 to P10 is unchanged (Figure 4D). As a result, we confirmed that the rCA16-Nluc virus is a genetically stable modified enterovirus.

### Antiviral assays of GuHCl, ribavirin, chloroquine and NH_4_Cl using rCA16-Nluc

**Figure 5:**
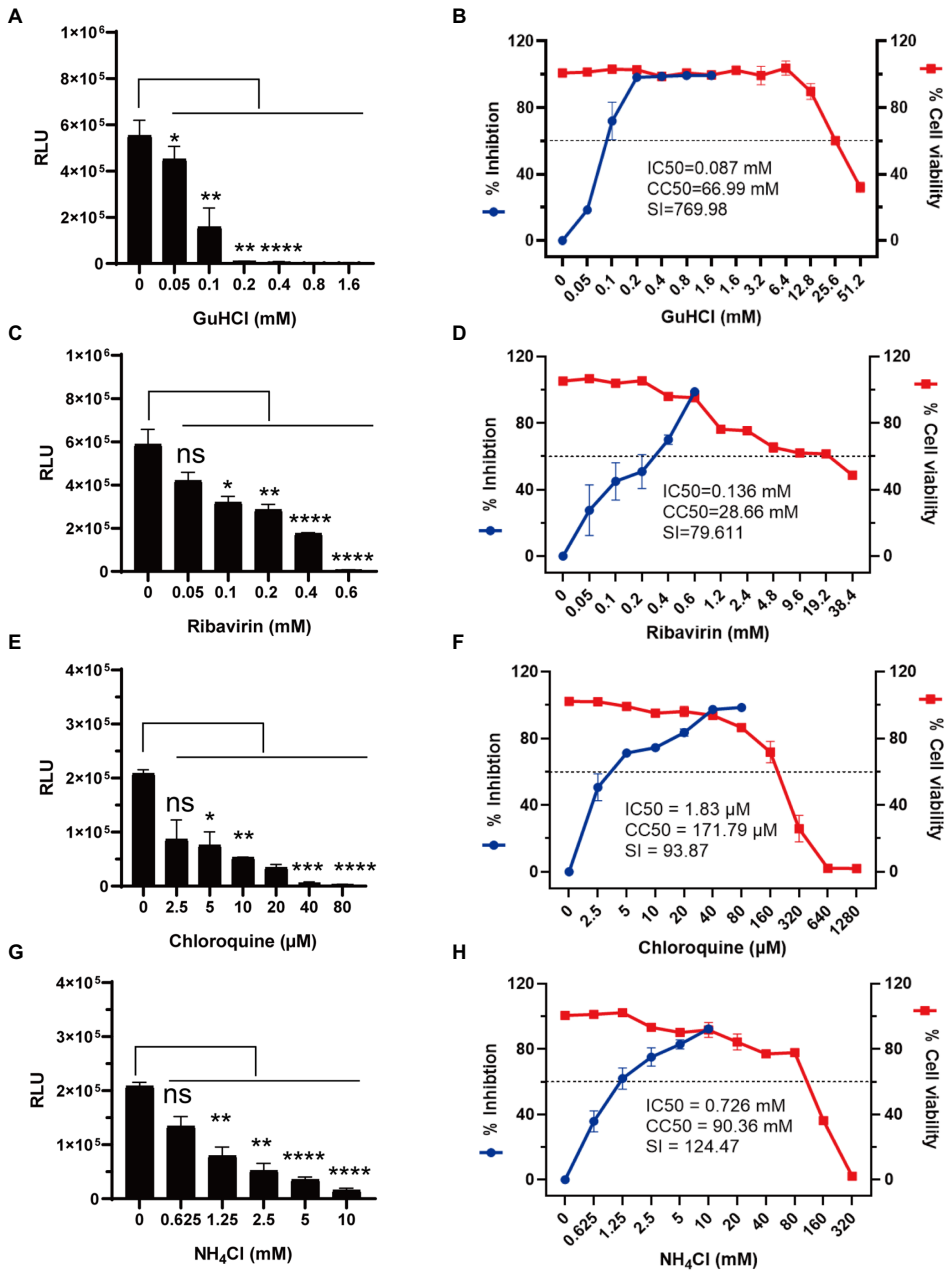
Antiviral testing of GuHCl, ribavirin, chloroquine and NH_4_Cl based on rCA16-Nluc. Vero cells were seeded in 96-well plates overnight before rCA16-Nluc (MOI = 0.1) inoculation and antiviral treatment as indicated. RLU was measured 24 h afterwards. Cell viability was measured by the CCK-8 assay. **(A,B)** show the luminescence, the calculated inhibition rates and cell viability under different concentrations of GuHCl. **(C,D)** show the luminescence, the calculated inhibition rates and cell viability under different concentrations of ribavirin. **(E,F)** show the luminescence, the calculated inhibition rates and cell viability under different concentrations of chloroquine. **(G,H)** show the luminescence, the calculated inhibition rates and cell viability under different concentrations of NH_4_Cl. Statistical differences were determined using t-test (*P < 0.05; **P < 0.01; ***P < 0.001; ****P < 0.0001).

Luciferase has a great advantage over fluorescent protein as the reporter gene in high-throughput screening assays. To see if the rCA16-Nluc can be applied in the settings of high-throughput drug screening, we chose to test two viral replication inhibitors GuHCl and ribavirin (Murray and Nibert, 2007; Li et al., 2008), and two endosomal acidification inhibitors chloroquine and NH_4_Cl (Lee et al., 2014; Tan et al., 2018). Indeed, we found that the increased concentrations of the tested inhibitors increasingly inhibited luciferase activity in Vero cells infected with rCA16-Nluc (Figures 5A,C,E,G). Based on this assay, the IC_50_ of GuHCl, ribavirin, chloroquine, and NH_4_Cl were calculated to be 0.07 mM, 0.136 mM, 1.83 μM, and 0.726 mM, respectively, (Figures 5B,D,F,H). Besides, we also measured the CC_50_ and SI index of each antiviral inhibitor. Together, we have demonstrated that rCA16-Nluc is more cost-effective and faster, and has great application value in high throughput drug screening.

### Using rCA16-Nluc in neutralizing antibody assays

**Figure 6:**
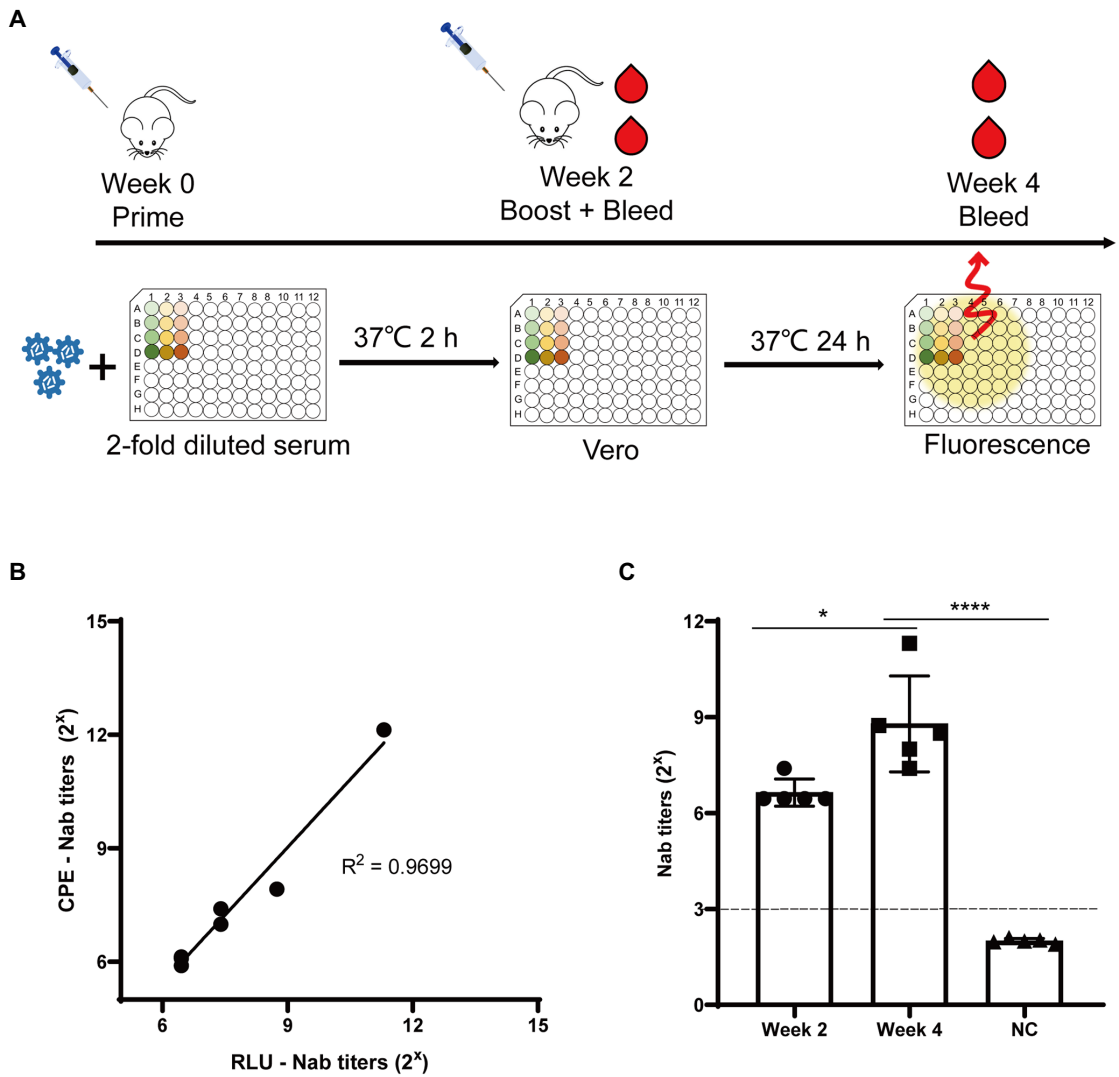
Luminescence-based tests for CA16 neutralizing antibodies. **(A)** Schematic diagram of the immunization procedure and analysis of neutralization antibodies in mice. **(B)** Correlation between the traditional neutralizing antibody test (CPE-Nab) and the luciferase-based neutralizing antibody test (RLU-Nab). **(C)** Serum neutralizing antibody titers at second and fourth week post primary immunization. Statistical differences were determined using t-test (*P < 0.05; **P < 0.01; ***P < 0.001; ****P < 0.0001).

Neutralizing antibodies are widely used to prevent and treat viral infections. The neutralizing antibody titer is classically calculated according to the Reed-Muench algorithm analyzing the formation of CPE, called CPE-Nab assay here. However, this method is not suitable for titration of a large number of samples because it is labor-intensive and cumbersome. Thus, we developed a simple assay (called RLU-Nab assay) to measure neutralizing antibody titers based on the rCA16-Nluc reporter virus. To this end, the anti-CA16 serum was produced by immunizing the C57/B6 mice twice with CA16 as shown in Figure 6A. Then the mouse serums were collected 2 or 4 weeks after the virus challenge, and tested for CA16 neutralizing activity. The neutralizing antibody titers measured in both tests are highly consistent (*R*^2^ = 0.9699, Figure 6B), suggesting a high quality of the RLU-Nab assay. Our data also showed that the neutralizing antibody titers after the second viral challenge were significantly higher than those of the first challenge (Figure 6C). Thus, the RLU-Nab assay based on rCA16-Nluc is an easy method and has the potential to be used in high-throughput antibody tests.

## Discussion

In this report, we describe the development and characterization of a novel CA16 infectious cDNA clone which produced the recombinant CA16 virus (OP293089). The virological features of rCA16 is comparable to its parental pCA16, for both plaque morphology and replication kinetics. Besides, rCA16 infection (10^5^ TCID_50_ viruses i.p.) in one-week-old lactating mice is 100% lethal and highly pathogenic causing serious damage of multiple tissues, including the brain, hearts, lungs and muscles. In parallel with the fact that majority of enterovirus-infected HFMD cases are under 5 years of age (Sharma et al., 2022), neonatal mouse or young animals is usually required for establishing the pathogenic enterovirus infection model (Zhang et al., 2019; Chen Y. et al., 2021; Li et al., 2022). Previous studies have shown that gerbils younger than 3-weeks were fully susceptible to infection by a clinical CA16 isolate and all died within 5 days of infection at a TCID_50_ of 10^5.5^ (Sun et al., 2016). Thus, the gerbil is considered to be a good model of small animals in CA16 pathogenesis studies, vaccine development and drug evaluation (Sun et al., 2022). Besides, Liu et al. have reported that infection with 2 * 10^6^ TCID_50_ CA16 infection is fatal in one-day-old BALB/c mice (Liu Q. et al., 2014), while the intracerebral infection with BJCA08/CA16 isolate in one-day-old ICR mice showed a LD50 as 1.2 PFU/mouse (Mao et al., 2012). Here, we have shown that OP293089 CA16 strain is highly pathogenic even in one-week-old mice, which will greatly facilitate future pathogenesis studies. Furthermore, the creation of the infectious clone of the same CA16 strain will provide additional values to the animal models, given that it will make it possible to further explore of the viral genome to identify potential pathogenic mechanisms.

Reporter viruses enabled convenient virus tracing and quantitative analyses which are required for high-throughput screening assays (Whitehead et al., 1979; Hoenen and Feldmann, 2014). A number of human enterovirus infectious clones with various reporter genes have been reported. For example, Deng et al. created a eGFP-CA16 infectious clone whose progeny produced smaller plagues (Deng et al., 2015). Besides, eGFP-EV71 and DsRed-EV71 infectious clones have also been reported (Shang et al., 2013). When compared, the recombinant viruses with fluorescent protein reporters are not as suitable as those with luciferase reporters in quantitative and high-throughput analyses, Yun et al. reported two recombinant Zika viruses expressing eGFP-or Nanoluc-reporter respectively, nevertheless only the ZIKA-Nluc was adopted to further evaluate antivirals (Yun et al., 2020). Nanoluc luciferase has a higher luminescence intensity, a lower background, and is more stable in variable pH and temperatures than Firefly luciferase and Renilla luciferase (Hall et al., 2012). These features make the Nluc reporter gene more suitable for high-throughput testing of antivirals and neutralizing antibodies (Chen K. Y. et al., 2021). Therefore, by inserting a Nanoluc luciferase gene between the 5′UTR and VP4 of CA16 genome, we successfully constructed the rCA16-Nluc infectious clone and rescued the viable recombinant virus. The use of rCA16-Nluc enabled us to measure conveniently the antiviral SI index for guanidine hydrochloride, ribavirin, chloroquine, and NH_4_Cl. Moreover, it also allowed the development of a rapid and efficient quantitative testing of neutralizing antibodies against CA16. These results indicate that the rCA16-Nluc virus has great potential for high-throughput screening.

The genetic stability of reporter gene in recombinant enterovirus is a difficult issue since the reporter gene can be easily and quickly lost through recombination. It has been reported that the eGFP-EV71 virus would lose the reporter gene up to P5 passage (Shang et al., 2013), while the stability of a previously established eGFP-CA16 is not reported and unlikely stable (Deng et al., 2015). Comparing with other reporters, the Nluc gene encodes a 19 kDa luciferase, which is the smallest member among Gaussia luciferase (19.9 kDa) and Firefly luciferase (41 kDa; Hall et al., 2012). Since smaller size may contribute to higher genetic stability. Nluc has been inserted to an EV71 infectious clone and this EV71-Nluc virus is genetically stable and confers stable luciferase activity over 10 passages in cultured cells (Yang et al., 2021), overcoming its shorter lifetime in EV71-Gluc virus (Xu et al., 2015). In another reverse genetic study of Senecavirus A with a insertion of Nluc gene between 2A and 2B, this reporter virus is also stable over 10 passages in BHK-21 cells (Guo et al., 2021). Consistent with these earlier reports, we report here that the rCA16-Nluc virus has been confirmed stable in Vero cells by multiple assays. To our knowledge, this is the first reported CA16 infectious clone with a Nluc reporter and the higher genetic stability of the rCA16-Nluc clone is crucial for its future application.

In summary, we have established the two new CA16 infectious clones with great application potential. The rCA16 virus showed a high pathogenicity in one-week-old mice, while the rCA16-Nluc virus is genetically stable and can be used in high-throughput screening testing for antivirals and neutralizing antibodies.

## Data availability statement

The original contributions presented in the study are included in the article/Supplementary material, further inquiries can be directed to the corresponding authors.

## Ethics statement

The animal study was reviewed and approved by Shanghai Public Health Clinical Center Laboratory Animal Welfare and Ethics Committee (Approval number: 2022-A030-02).

## Author contributions

RY: methodology, conducting experiment, data collection, and writing–original draft. MW, LL, and JY: providing key reagent. JF, XL, and MK: data analysis. JX and XZ: conceptualization, supervision, and funding acquisition. SZ: conceptualization, supervision, project administration, writing–review and editing, and funding acquisition. All authors contributed to the article and approved the submitted version.

## Funding

This project was supported by the National Natural Science Foundation of China (grant nos. 82172250 and 82002135) and Shanghai Public Health Clinical Center (grant nos. KY-GW-2020-10).

## Conflict of interest

The authors declare that the research was conducted in the absence of any commercial or financial relationships that could be construed as a potential conflict of interest.

## Publisher’s note

All claims expressed in this article are solely those of the authors and do not necessarily represent those of their affiliated organizations, or those of the publisher, the editors and the reviewers. Any product that may be evaluated in this article, or claim that may be made by its manufacturer, is not guaranteed or endorsed by the publisher.
