# Supplementary material for: The development and characterization of a stable Coxsackievirus A16 infectious clone with Nanoluc reporter gene
Source: Front Microbiol. 2023 Jan 10;13:1101850. doi: 10.3389/fmicb.2022.1101850 (PMC9871592; doi:10.3389/fmicb.2022.1101850)

Supplementary Material for “The development and characterization of a stable Coxsackievirus A16 infectious clone with NanoLuc reporter gene”

**Rui Yu^1^, Min Wang^1^, Lizhen Liu^1^, Jingjing Yan^1^, Jun Fan^1^, Xiaohong Li^1^, Miaomiao Kang^1^, Jianqing Xu^1*^, Xiaoyan Zhang^1*^, Shuye Zhang ^2*^**


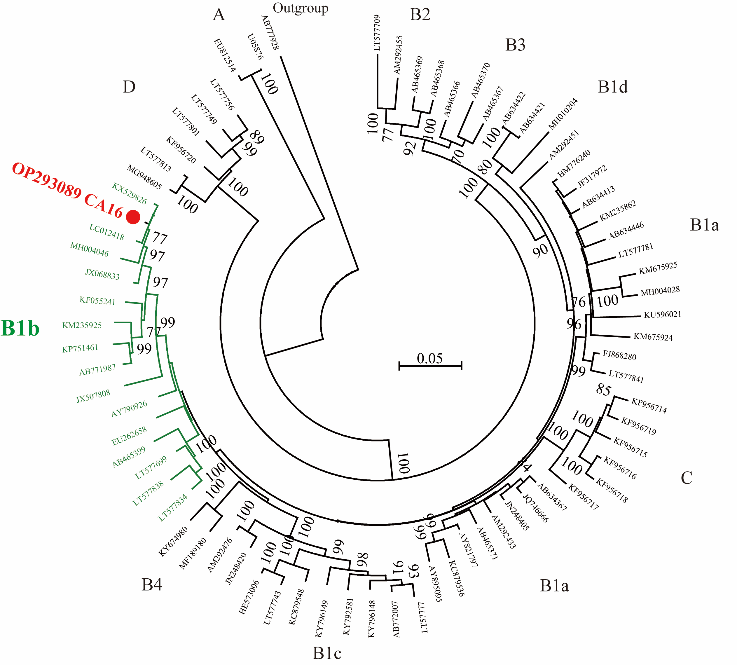


**Supplementary Figure 1.** Phylogenetic analysis of the CA16 strain (OP293089).


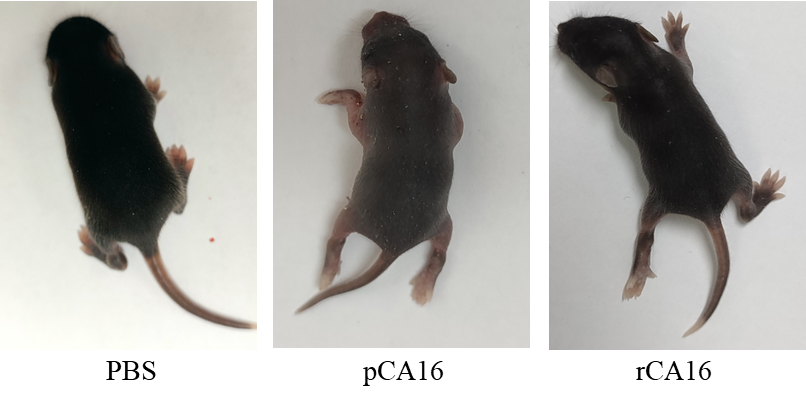


**Supplementary Figure 2.**  Paralysis in neonatal mice infected with the 10^5^ TCID_50_ pCA16 and rCA16 viruses 3 days after infection, mice injected with an equal volume of PBS as the control group.


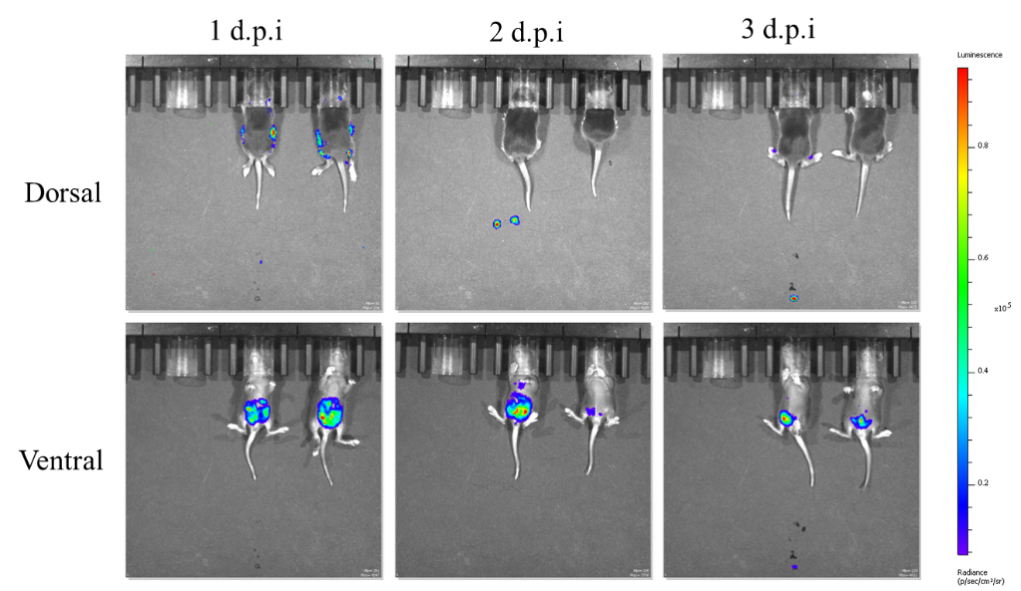


**Supplementary Figure 3.** One-week-old C57/B6 mice were infected with 10^5^ TCID_50_ rCA16-Nluc virus, and the bioluminescence intensity was analyzed by *in vivo* imaging on the first, second and third days after infection. A significantly higher radiance was detected at 1 d.p.i.

**Supplementary Table 1**

The PCR parameters of Nluc gene.


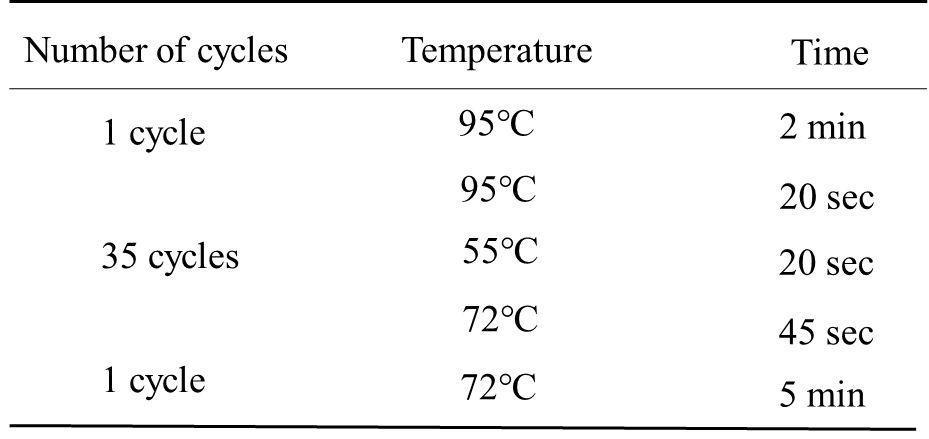


**Supplementary Table 2**

The primers for Nluc PCR and sequencing.


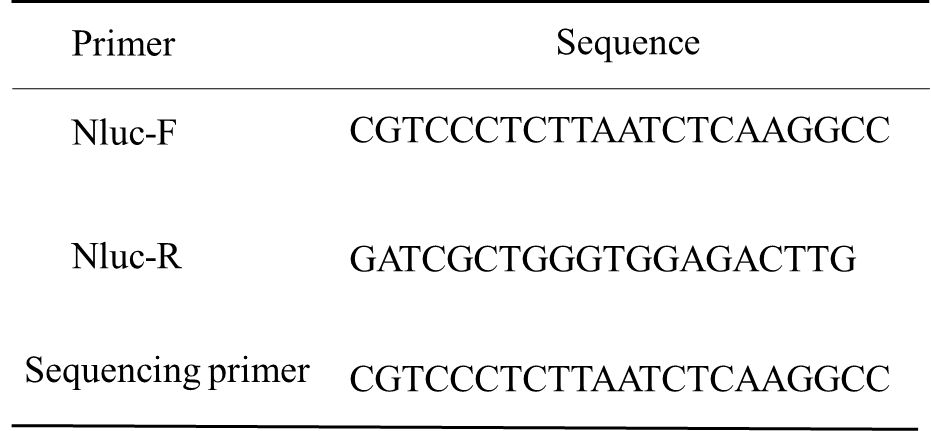


**Supplementary Table 3**

The primers for constructing the two infectious clones. We used the seamless cloning strategy (Hieff Clone® Plus One Step Cloning Kit, https://www.yeasen.com/products/detail/818, based on homologous recombination principle) to construct the infectious clones. For constructing the CA16 infectious clone: firstly, the primers (P1-F, P1-R; pcDNA-F, pcDNA-R) were used to amplify the P1 fragment and pcDNA vector for constructing of pcDNA-P1; the primers (P2-F, P2-R; P3-F, P3-R; pUC57-F, pUC57-R) were used to amplify the P2 fragment, P3 fragment and pUC57 vector for constructing of pUC57-P2+P3. Then the primers (P1-full-F, P1-full-R; P2+P3-full-F, P2+P3-full-R; pSVA-F, pSVA-F) were used to amplify the P1 fragment, P2+P3 fragment and pSVA vector for constructing of pSVA-CA16 infectious clone.

The CA16-Nluc infectious clone was constructed by adding Nluc gene to the CA16 infectious clone, the primers (CA16-Nluc-F, CA16-Nluc-R) were used to amplify the Nluc gene, and the primers (CA16-F, CA16-R) were used to amplify the CA16 infectious clone, and the CA16-Nluc infectious clone was constructed by recombination of the two fragments.


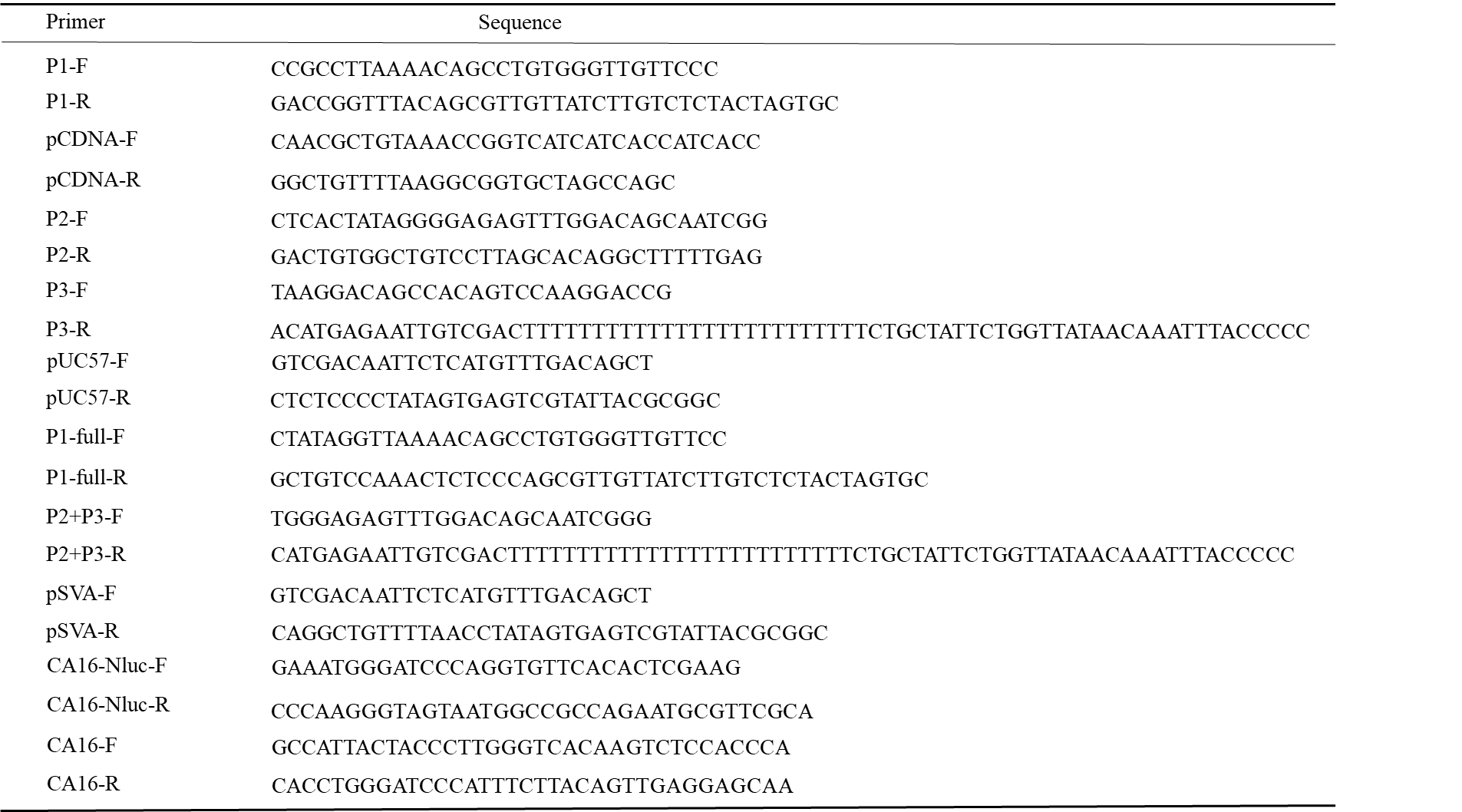

Supplement: Supplementary file 1 [file Data_Sheet_1.docx]
